# Moraxella catarrhalis infection participates in Alzheimer's disease via APP coupling and Aβ production through the outer membrane protein UspA1

**DOI:** 10.1002/alz70855_101378

**Published:** 2025-12-23

**Authors:** Jingxian Chen, Bin Jiao, Lu Shen

**Affiliations:** ^1^ Xiangya Hospital, Central South University, Changsha, Hunan, China

## Abstract

**Background:**

Alzheimer's disease (AD), the most common form of dementia, has been linked to various hypotheses, including the Aβ cascade, tau protein hyperphosphorylation, and microbial inflammatory responses. However, the role of oral microbiota in AD pathogenesis remains unclear.

**Methods:**

Using the Illumina Novaseq 6000 platform for 16S rDNA sequencing and qPCR, the detection rate and expression of *Moraxella catarrhalis* were compared in the cerebrospinal fluid of 108 AD patients (based on the 2018 ATN framework) and 46 controls. To explore its role in AD pathogenesis, SY5Y‐APPswe and SH‐SY5Y cells were treated with varying concentrations of *M. catarrhalis*, pasteurized *M. catarrhalis*, outer membrane vesicles (OMVs), and UspA1 overexpression, with APP and Aβ expression analyzed via Western Blot and ELISA. In 5xFAD mouse models, *M. catarrhalis* and saline were administered intranasally, while PKH6‐labeled OMVs and AAV‐UspA1‐EGFP were stereotactic injected into the hippocampus, followed by behavioral and pathological assessments.

**Results:**

The relative abundance of Moraxella species was significantly higher in the AD group, suggesting a potential association with AD pathogenesis. At the cellular level, we found that intervention with Moraxella catarrhalis and its extracellular vesicles, as well as overexpression of UspA1 protein, significantly upregulated APP and extracellular Aβ levels. In animal experiments, after Moraxella catarrhalis intervention or overexpression of UspA1 protein in the bilateral hippocampus of AD model mice, APP and Aβ protein levels in the cortical and hippocampal regions of the brain were significantly elevated, and learning, memory, and cognitive impairments were exacerbated.

**Conclusions:**

This study suggests that Moraxella catarrhalis contributes to AD pathogenesis through its extracellular vesicles and the UspA1 outer membrane protein, which participate in the APP proteolytic pathway and Aβ generation.

